# Cannabinoid-2 Receptor Activation Attenuates Sulfur Mustard Analog 2-Chloroethyl-Ethyl-Sulfide-Induced Acute Lung Injury in Mice

**DOI:** 10.3390/ph18020236

**Published:** 2025-02-10

**Authors:** Gregory Nicholson, Nicholas Richards, Janette Lockett, My Boi Ly, Raj V. Nair, Woong-Ki Kim, K. Yaragudri Vinod, Nagaraja Nagre

**Affiliations:** 1 Department of Biomedical and Translational Sciences, Macon & Joan Brock Virginia Health Sciences, Eastern Virginia Medical School at Old Dominion University Norfolk, Old Dominion University, Norfolk, VA 23507, USA; g.lnicholson@yahoo.com (G.N.); richarnc@odu.edu (N.R.); meipei1908@gmail.com (M.B.L.); nairrv@odu.edu (R.V.N.); 2 Department of Microbiology & Immunology, Tulane University School of Medicine, Tulane University, New Orleans, LA 70112, USA; wkim6@tulane.edu; 3 Division of Microbiology, Tulane National Primate Research Center, Tulane University, Covington, LA 70433, USA; 4 Emotional Brain Institute, Nathan Kline Institute for Psychiatric Research, Orangeburg, NY 10962, USA; vinod.yaragudri@nki.rfmh.org; 5 Department of Child & Adolescent Psychiatry, New York University Langone Health, New York, NY 10016, USA

**Keywords:** cannabinoids, cannabinoid receptor-2, 2-chloroethyl ethyl sulfide, acute lung injury, C57BL/6 J mice

## Abstract

**Background:** Exposure to sulfur mustard (SM; 2,2′-dichlorodiethyl sulfide) causes toxicity in the human body, particularly the lungs. The molecular mechanisms of SM-induced lung damage are elusive, and no effective treatments exist. This study explores the anti-inflammatory potential of cannabinoid receptor 2 (CB2R) activation in mitigating acute lung injury (ALI) and inflammation induced by 2-chloroethyl ethyl sulfide (CEES), a structural analog of SM. **Methods:** C57BL/6J mice were exposed to CEES via intratracheal administration to model ALI. CB2R activation was achieved through the intraperitoneal administration of HU308, a selective synthetic agonist. ALI and inflammation were evaluated at 48 h post-exposure to CEES. Bronchoalveolar lavage fluid (BALF) was collected to measure total cells, protein, and cytokines. Lung injury, inflammatory signaling in alveolar macrophages (AMs), and matrix metalloproteinase-9 (MMP-9) activity were assessed via histological analysis, immunoblotting, and gelatin zymography, respectively. **Results:** CEES exposure led to an increase in immune cell infiltration, pro-inflammatory cytokines (IL-6 and TNF-α), and pro-MMP9 levels in the BALF, which were significantly decreased by HU308 treatment. The activation of CB2R attenuated CEES-induced NF-κB activation and reduced pro-inflammatory M1 markers (iNOS, and Cox-2) but did not alter the increase in the M2 marker arginase-1. CB2R activation mitigated CEES-induced oxidative stress, as evidenced by lower levels of heme oxygenase-1 (HO-1) and reactive oxygen species (ROS) in mouse AMs. Additionally, 4-hydroxynonenal (4-HNE) levels were reduced in the lungs of HU308-treated mice but were elevated after CEES exposure. **Conclusions:** These findings suggest that CB2R activation alleviates CEES-induced ALI and inflammation in mice, supporting its potential as a therapeutic approach for vesicant-induced pulmonary injury.

## 1. Introduction

Sulfur mustard (SM), a well-known blistering agent from the mustard gas family, remains a significant concern due to its historical use in chemical warfare, existing stockpiles, and ease of synthesis, which collectively increase the potential for intentional misuse or accidental exposure [1]. The damage to the respiratory, ocular, and cutaneous systems caused by SM, as well as the resulting infections, is usually fatal due to the absence of effective treatments [2,3]. The pathophysiology of human cases and experimental animal models has shown that the inhalation of SM causes direct insult to the lung leading to injury and acute effects, like acute respiratory distress syndrome (ARDS), increased inflammation, hypoxia, and impaired gas exchange [4,5]. Acute exposure to SM impacts both the upper and lower airways, triggering airway hyperreactivity, pulmonary edema, activation of the coagulation cascade, and the formation of airway fibrin casts, ultimately resulting in acute respiratory failure and death [6,7]. The chronic complications of SM involve chronic bronchitis, bronchopneumonia, and lung fibrosis [8,9].

Similar pathological effects on the lungs have been reported to be induced by a mono-functional analog of SM, 2-chloroethyl ethyl sulfide (CEES) [10,11]. The exposure of lung tissue to CEES induces the significant accumulation of inflammatory exudate and an increased cellular infiltration within the alveoli during the acute phase [12,13]. Alveolar macrophages are the key cell types that mediate vesicant-induced lung inflammation in rodents. Prolonged activation of the M1 (proinflammatory/cytotoxic) phenotype of macrophages leads to the excessive release of inflammatory mediators and acute lung injury [14,15]. Another catastrophic effect of CEES toxicity is the oxidative stress contributing to a disturbance in the redox balance, direct depletion of cellular thiols, such as glutathione (GSH), alkylation or oxidation of DNA strands, lipid peroxidation, and an overproduction of reactive oxygen species (ROS). The oxidative stress further leads to the deterioration of extracellular matrix remodeling, apoptosis of alveolar cells, and impairment of mitochondrial respiration resulting in lung tissue damage [15,16]. Such a disintegration of the cellular system demands novel strategies to counteract the inflammation and oxidative stress induced by these vesicants.

The mammalian endocannabinoid system (ECS) plays a pivotal role in various physiological functions and comprises cannabinoid (CB) receptors, endocannabinoids, such as N-arachidonoyl ethanolamine (anandamide/AEA) and 2-arachidonoylglycerol (2-AG), and the enzymes responsible for their synthesis and degradation [17,18]. AEA and 2-AG exert their effects primarily by binding to two G-protein coupled receptors (GPCRs), CB1 receptors (CB1R) and CB2 receptors (CB2R), respectively [19,20,21]. While CB1R is predominantly expressed in the brain, CB2R is mainly localized in the peripheral system, where it plays a key immunomodulatory role [22,23]. The activation of CB2R has been shown to exhibit anti-inflammatory and anti-fibrogenic effects without inducing adverse psychotic outcomes. As a result, the role of CB2R in immune regulation has garnered significant therapeutic interest [24]. The pharmacological activation of CB2R in the lungs has demonstrated protective effects in models of paraquat-induced acute lung injury in rats and bleomycin-induced pulmonary fibrosis in mice [25,26]. Additionally, the activation of CB2R through the elevation of 2-AG attenuates the lipopolysaccharide (LPS)-induced acute lung injury in mice [27]. Research from our laboratory has demonstrated that the pharmacological activation of CB2R attenuates acute lung injury and inflammation induced by bacterial pneumonia in a mouse model [28]. However, the impact of CB2R signaling in vesicant-induced lung injury remained unexplored. Our study aims to investigate the role of CB2R signaling in vesicant-induced acute lung injury, an area that remains unexplored despite prior evidence of CB2R’s protective effects in other lung injury models. In this study, we utilized an SM analog, CEES, to induce acute lung injury in mice.

We have shown that the activation of CB2R with the selective synthetic agonist HU308 effectively mitigates CEES-induced lung injury and inflammation. Additionally, HU308 significantly reduced the oxidative stress associated with CEES exposure. Furthermore, CB2R activation was found to suppress the activation of NF-κB and reduce activation of the M1 (proinflammatory/cytotoxic) phenotype of macrophages.

## 2. Results

### 2.1. CB2R Activation Alleviates CEES—Induced Lung Injury and the Decline in Lung Function

**Figure 1:**
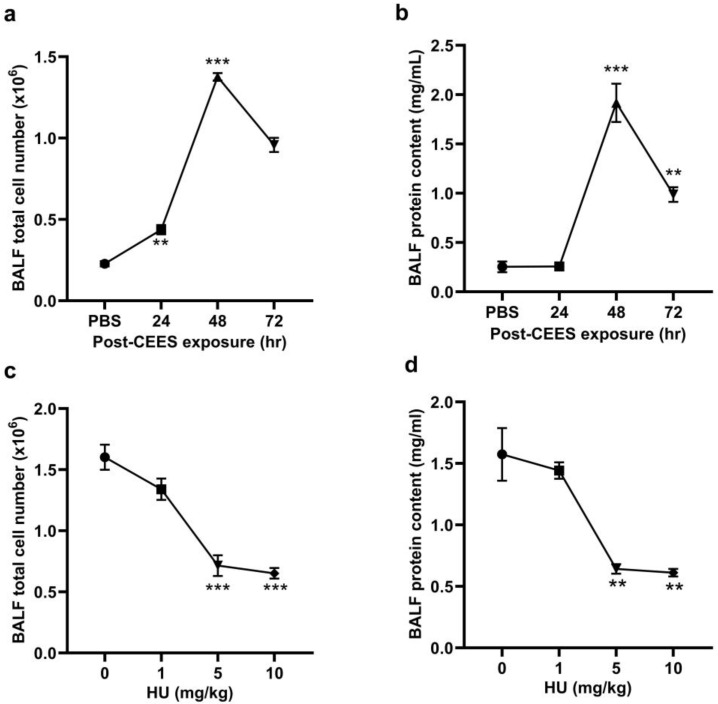
C57BL/6J WT mice were exposed to CEES (6 mg/kg). The total cell number (**a**) and protein content (**b**) in the BALF were determined at 24, 48, and 72 h post-CEES exposure. The total cell number (**c**) and total protein content (**d**) in the BALF were determined from the mice treated with varying doses of HU308 and exposed to CEES. *n* = 6, ** *p* < 0.01, *** *p* < 0.001. Data are presented as the mean ± SEM. HU: HU308.

CEES-induced lung injury was modeled in mice using the intratracheal (i.t.) administration of CEES. Lung injury and inflammation were assessed at 24, 48, and 72 h post-exposure to determine the optimal time point for achieving reproducible acute lung injury. The total cell number and protein content in BALF were measured at these time intervals. The highest increase in both BALF cell numbers (Figure 1a) and protein content (Figure 1b) was observed at 48 h post-CEES exposure. Consequently, the 48-h time point was selected for subsequent experiments. To activate the CB2R, HU308 was administered intraperitoneally (i.p.) to mice 1 h before exposure to CEES, with additional doses given every 24 h after that. Various doses of HU308 (1, 5, and 10 mg/kg body weight) were tested to identify the optimal dosing regimen. Total BALF cell counts and protein contents were assessed 48 h post-CEES exposure. The administration of 5 mg/kg HU308 significantly reduced both the total cell number (Figure 1c) and protein content in the BALF (Figure 1d), and this dosage was utilized in subsequent experiments.

**Figure 2:**
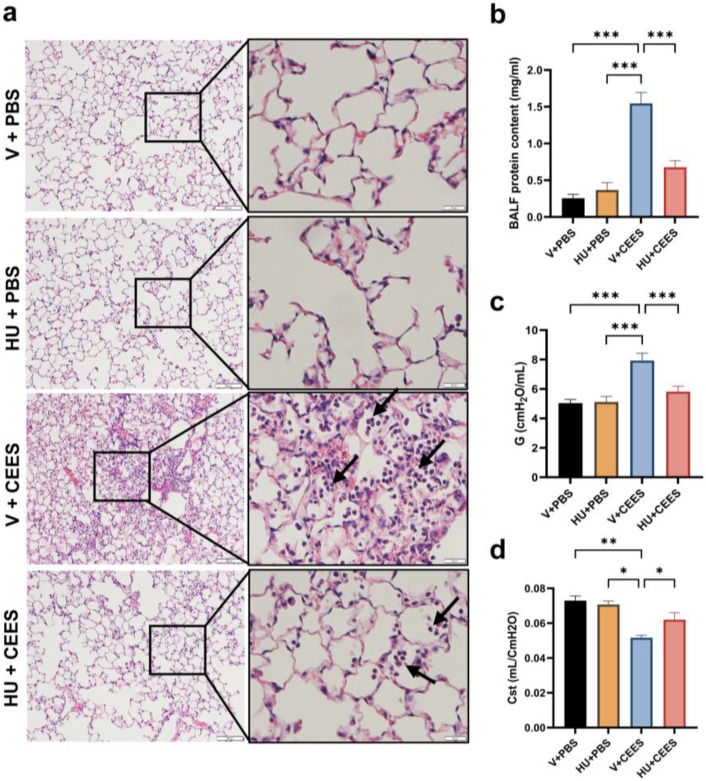
CB2R activation mitigates CEES-induced lung damage and preserves lung function. C57BL/6J WT mice received either vehicle or HU308 and were treated with PBS (control) or CEES. Lung injury was assessed 48 h post-treatment. (**a**) Representative H&E-stained lung tissue images, with low magnification (scale bar: 100 µm) and high magnification (scale bar: 20 µm). The black arrows are pointing at immune cells. (**b**) Total protein levels in bronchoalveolar lavage fluid (BALF) were measured (*n* = 6). (**c**) Tissue dampening (G) and (**d**) static lung compliance (Cst) were evaluated using the FlexiVent System with FlexiWare software. *n* = 5, * *p* < 0.05, ** *p* < 0.01, *** *p* < 0.001. Data are presented as the mean ± SEM. V: vehicle, HU: HU308.

Mice exhibited typical lung injury and immune cell infiltration at 48 h following CEES instillation. A histological analysis of hematoxylin and eosin (H&E)-stained lung sections showed increased inflammatory cell infiltration in vehicle-treated mice after CEES exposure. Treatment with HU308 reduced these histopathological changes (Figure 2a). HU308-treated mice also showed a reduction in BALF protein levels, a marker of lung microvascular permeability (Figure 2b). A further evaluation of lung mechanics using the FlexiVent rodent ventilator and FlexiWare software revealed an increase in parenchymal resistance (G) and a decrease in static lung compliance (Cst) in response to CEES exposure. In contrast, HU308 treatment notably attenuated the CEES-induced decline in lung function (Figure 2c,d).

### 2.2. CB2R Activation Alleviates Lung Inflammation Induced by CEES

**Figure 3:**
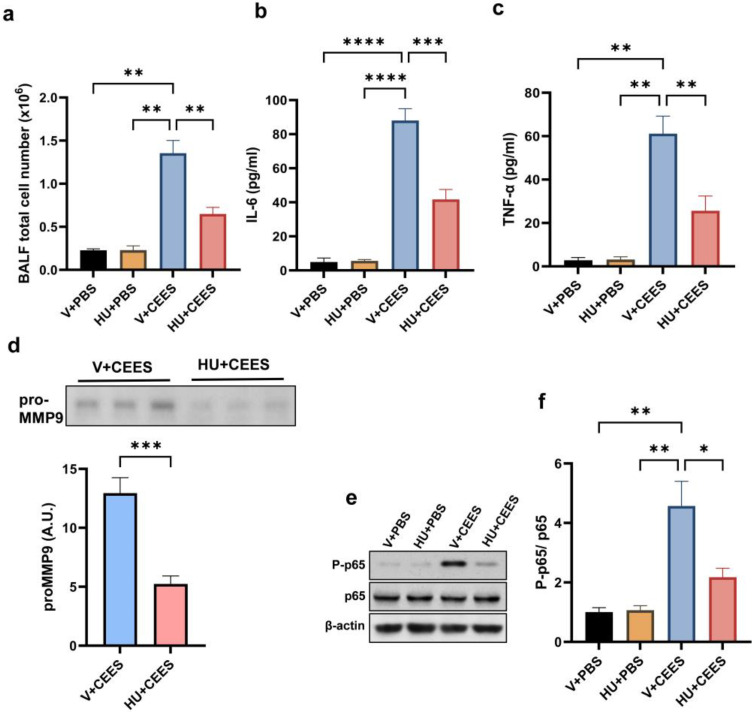
HU308 treatment alleviates CEES-induced pulmonary inflammation in mice. C57BL/6J WT mice were treated with either vehicle or HU308 and exposed to PBS (control) or CEES. After 48 h, BALF was collected for analysis. Total cell counts (**a**) were determined (*n* = 5), and inflammatory cytokine levels, IL-6 (**b**) and TNF-α (**c**), were measured (*n* = 6). Additionally, pro-MMP-9 levels in the BALF were evaluated, with the representative data and quantification shown in panel (**d**) (*n* = 6). CB2R activation inhibits CEES-induced NF-κB activation, as demonstrated by assessing P-p65 and p65 protein levels in AMs. The representative immunoblot images are provided in panel (**e**), with the densitometry analysis presented in panel (**f**). *n* = 5–6, * *p* < 0.05, ** *p* < 0.01, *** *p* < 0.001, and **** *p* < 0.0001. Data are expressed as the mean ± SEM. V: vehicle, HU: HU308.

Immune cell infiltration into the lungs was evaluated by measuring the total cell count in the BALF. The activation of CB2R significantly reduced immune cell infiltration, as evidenced by a lower BALF total cell count in HU308-treated mice compared to vehicle-treated mice (Figure 3a). Further, we observed that CEES exposure led to an increase in the levels of IL-6 and TNF-α in the BALF. Interestingly, HU308-treated mice had significantly lower BALF IL-6 (Figure 3b) and TNF-α (Figure 3c). MMP-9 activity in the BALF was assessed through gelatin zymography. Our results showed that CEES exposure resulted in a significant increase in pro-MMP-9 levels in the BALF. However, HU308-treated mice had markedly lower pro-MMP-9 in the BALF (Figure 3d). NF-κB is a key mediator of the inflammatory response and principal transcriptional activator of proinflammatory cytokines. Hence, investigating the effects of CB2R activation induced by HU308 on CEES-induced NF-κB activation is of critical importance. An immunoblot analysis of AMs demonstrated the upregulation of phosphorylated p65 (P-p65) levels, which indicates NF-κB activation, following CEES exposure in mice. Conversely, treatment with HU308 significantly reduced P-p65 levels (Figure 3e,f). These findings suggest that the activation of CB2R attenuates the CEES-induced upregulation of inflammatory cytokines by downregulating NF-κB signaling pathways.

### 2.3. HU308 Treatment Suppresses CEES-Induced Proinflammatory Macrophage Phenotype

**Figure 4:**
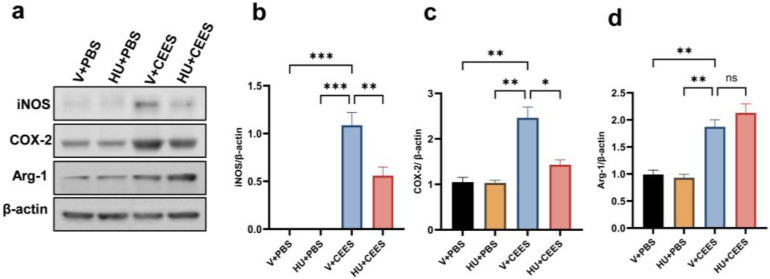
CB2R activation reduces the CEES-induced proinflammatory macrophage phenotype. C57BL/6J WT mice were treated with vehicle or HU308 and exposed to either PBS (control) or CEES. The macrophage M1 phenotype was examined by measuring the levels of iNOS and Cox-2 in AMs. Representative immunoblot images are displayed in panel (**a**), while the corresponding densitometry data are presented in panels (**b**) and (**c**). The protein level of the M2 macrophage marker was also examined. A representative immunoblot image is shown in panel (**a**) and densitometry data are presented in panel (**d**), *n* = 5, * *p* < 0.05, ** *p* < 0.01, *** *p* < 0.001. ns: non-significant. Data are presented as the mean ±SEM. V: vehicle, HU: HU308.

Vesicant exposure triggers the activation and polarization of macrophages, contributing to acute inflammation [13,16]. We investigated the expression of proinflammatory, M1 macrophage markers in AMs. Our results demonstrated that the expression levels of inducible nitric oxide synthase (iNOS) and cyclooxygenase-2 (Cox-2) were significantly upregulated in response to CEES exposure. Notably, AMs from mice treated with HU308 exhibited markedly lower expression levels of iNOS and Cox-2 (Figure 4a–c). Additionally, we observed an increase in the M2 macrophage marker arginase-1; however, CB2R activation did not alter these changes (Figure 4a,d). Collectively, these findings suggest that CB2R activation by HU308 suppresses proinflammatory macrophages, thereby mitigating CEES-induced acute lung injury.

### 2.4. Pharmacological Activation of CB2R Attenuates the CEES-Induced Oxidative Stress

**Figure 5:**
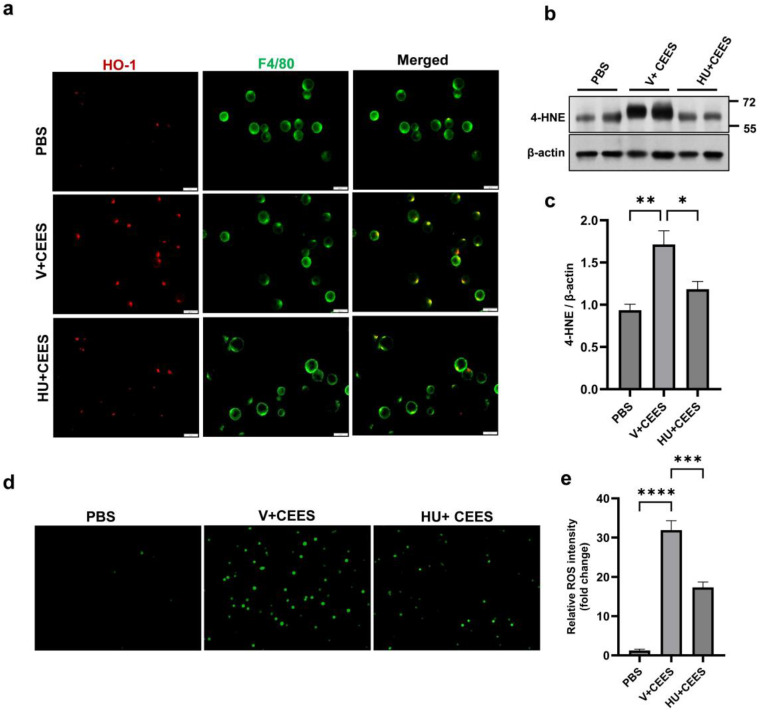
HU308 treatment reduces CEES-induced oxidative stress. AMs were isolated from WT mice, cultured, and exposed to CEES for 4 h. (**a**) Representative immunostaining image of HO-1 (red) in AMs. F4/80 staining (green) was used to indicate AMs. C57BL/6J WT mice were exposed to either PBS (control) or CEES with or without HU308 treatment, and 4-HNE levels in the lung were examined via immunoblotting. A representative immunoblot image is shown in panel (**b**), and densitometry data are presented in panel (**c**). *n* = 5. Representative DCFH-DA staining of ROS (**d**) and quantitative analysis (**e**) in AMs exposed to CEES with or without HU308 treatment. *n* = 5–6, * *p* < 0.05, ** *p* < 0.01, *** *p* < 0.001, **** *p* < 0.0001. Data are presented as the mean ± SEM. V: vehicle, HU: HU308.

CEES exposure is known to induce oxidative stress in the lung [15]. We measured the expression of HO-1 in mouse primary AMs following CEES exposure. CEES treatment resulted in increased HO-1 expression, while CB2R activation by HU308 reduced it (Figure 5a). Another indicator of oxidative damage is the formation of lipid peroxidation products, including 4-HNE, an unsaturated aldehyde, and a major byproduct generated during lipid peroxidation [29]. A marked increase in 4-HNE levels was observed in the lung tissue 48 h post-exposure to CEES. However, mice treated with HU308 exhibited significantly lower 4-HNE levels (Figure 5b,c), suggesting that CB2R activation mitigates the CEES-induced increase in lipid peroxidation. Further, we measured intracellular ROS in mouse primary AMs using a DCF fluorescent indicator. CEES exposure led to an increase in intracellular ROS, evidenced by elevated DCFH-DA staining. However, the HU308 treatment significantly reduced the DCF fluorescence intensity, indicating that CB2R activation alleviates CEES-induced ROS generation (Figure 5d,e). Overall, these results demonstrate that CB2R activation significantly reduces CEES-induced oxidative stress.

## 3. Discussion

In this study, we examined the impact of the pharmacological activation of CB2R on CEES-induced acute lung injury and inflammation. Our findings demonstrate that the activation of CB2R using the selective synthetic agonist HU308 significantly alleviated CEES-induced lung injury and inflammation. Furthermore, HU308 treatment effectively mitigated the heightened oxidative stress triggered by acute CEES exposure.

Sulfur mustard, a potent vesicant used against humans, continues to be recognized as a threat agent [3,4]. CEES (a.k.a. half-mustard), used in this study, is a surrogate of SM because of its structural and reactive group similarities to SM and because it mimics, to a large extent, the pathophysiology of animals and humans exposed to SM [10,11]. CEES-induced acute lung injury was modeled in WT mice, and the i.t. administration of CEES (6 mg/kg) produced quantifiable and reproducible lung injury in mice at 48 h. After testing various time points, it was observed that there is a notable increase in indicators of lung injury and inflammation at 48 h after exposure to CEES. Consequently, the 48 h time point was used throughout the study.

The EC system consists of endocannabinoids, CB1R, and CB2R and the enzymes involved in the synthesis and degradation of endocannabinoids. The endocannabinoid signaling exerts a considerable influence on various physiological and pathological processes [17,18,19]. These effects encompass potentially beneficial anti-inflammatory and anti-injury responses [30]. CB2R, the peripheral receptor for cannabinoids, is mainly expressed in immune tissues, playing an important role in immunomodulation. CB2R was shown to modulate immune cell functions in both in vitro and in vivo models of inflammatory diseases [23,31]. The development and application of selective CB2R agonists hold substantial promise. Among the various ligands assessed for off-target activity, selectivity, balanced signaling, and pharmacokinetic profiles, HU308 has emerged as a leading candidate, exhibiting the most favorable functional properties [32]. Therefore, HU308 was used in this study to activate CB2R. The lung injury observed at 48 h after CEES exposure was reduced by CB2R activation. Previous research suggests that leukocytes and their inflammatory mediators contribute substantially to the pathogenic response to vesicant exposure [10,11,33,34]. In our model, the i.t. administration of CEES elevated the total cell count in the BALF. However, mice treated with HU308 exhibited a significantly reduced number of cells in the BALF, indicating a diminished influx of immune cells into the lung. Moreover, the levels of the pro-inflammatory cytokines IL-6 and TNF-α in the BALF were significantly decreased following CB2R activation by HU308. Notably, mice receiving HU308 treatment exhibited significantly lower MMP9 levels in the BALF. These findings collectively suggest that CB2R plays a pivotal role in modulating the inflammatory response to acute CEES exposure conditions, thereby mitigating exaggerated inflammation. Canonical pathways, such as STAT3 signaling, TREM1 signaling, and NF-κB1 signaling, were identified to be significantly enriched after vesicant exposure, aligning with their known roles in mediating the inflammatory responses to lung injury [35]. Mechanistic investigations into CEES-induced lung injury revealed that acute exposure to CEES activates NF-κB within the lung. The intratracheal administration of CEES in our mouse model, also led to NF-κB activation in AMs, which was significantly attenuated upon treatment with HU308. Owing to their remarkable plasticity, pulmonary macrophages can rapidly adopt different phenotypes in response to their surrounding stimuli [36]. Earlier studies have shown that there is a sequential accumulation of pro- or anti-inflammatory macrophages in the lungs, mediating vesicant-induced lung inflammation and injury. Proinflammatory/cytotoxic M1 macrophages are known to be involved in the acute inflammatory responses to pulmonary toxicants, including mustard vesicants. After exposure to nitrogen mustard (NM, another vesicant), a significant increase in the number of M1 macrophages was observed on day 3 [13,14]. A recent transcriptomic study showed the significant upregulation of *Nos2*, *Ptgs2*, *Ccl2*, and *Il1b* at 1-day postexposure to NM [35]. The upregulation of these genes is associated with an M1 proinflammatory macrophage phenotype. In agreement with the previous findings, we observed that acute exposure to CEES increased the pro-inflammatory M1 macrophage markers iNOS and Cox-2 and that the activation of CB2R significantly reduced the levels of iNOS and Cox-2. However, CB2R activation mediated by HU308 did not alter the CEES-induced increase in arginase-1. These findings suggest that CB2R activation reduces acute lung inflammation by decreasing the M1 phenotype without affecting the M2 phenotype.

Accumulating evidence also indicates that oxidative and nitrosative stresses contribute significantly to mustard lung injury and pathogenesis [15,33]. HO-1, a stress-inducible enzyme with both antioxidant and anti-inflammatory properties, has been identified as a crucial player in this context [37]. The research has further suggested that HO-1 might also influence the development of profibrotic M2 macrophages within the lung [38]. Concurrently, ROS and electrophilic species have been implicated in acute lung injury and inflammation resulting from vesicant exposure. An early example of the role of oxidative stress in SM-induced lung injury involved studies where antioxidant administration attenuated lung injury in rats exposed to CEES [39]. Human biopsies from SM-exposed individuals also revealed the increased expression of enzymes that elevate endogenous ROS levels, correlating with heightened oxidative stress markers in bronchoalveolar lavage fluid [40].

Earlier studies have demonstrated that the activation of CB2R exerts protective effects against tissue damage associated with inflammation and oxidative stress across various organs, including the lung, heart, liver, brain, and bladder. For instance, treatment with HU-308 has been shown to protect mice from cisplatin-induced renal injury by reducing NOX2 expression and subsequent oxidative stress [41]. Another study showed that CB2R activation mitigates lung ischemia–reperfusion injury (LIRI) by inhibiting oxidative stress, with NOX2 playing a role in CB2-mediated protection against LIRI in mice [42].

The aforementioned findings establish a solid foundation for discussing the role of CB2R activation in regulating CEES-induced oxidative stress. In the current study, we observed the upregulation of HO-1 expression in primary AMs from mice, following CEES exposure. Notably, the pharmacological activation of CB2R suppressed HO-1 expression. Additionally, an increase in cellular ROS was detected in response to CEES exposure, while HU-308 treatment significantly reduced ROS production. Oxidative stress can trigger lipid peroxidation, leading to the formation of reactive electrophilic lipid species that can covalently modify proteins, thereby altering their structure and function. Among these electrophilic lipid species, 4-HNE- a reactive α, β-unsaturated aldehyde, has garnered significant attention due to its generation during oxidative stress and the peroxidation of polyunsaturated fatty acids [29,43]. Studies in rodents exposed to CEES showed the upregulation of 4-HNE in the lungs [43]. We also observed a significant increase in 4-HNE levels post CEES, which was markedly attenuated by the activation of CB2R. Collectively, these results indicate that CB2 receptor activation plays a pivotal role in modulating CEES-induced oxidative stress.

## 4. Materials and Methods

### 4.1. Animals

C57BL/6 J (WT) mice were purchased from the Jackson Laboratory, Bar Harbor, ME, USA, and were housed in a sterile ventilated facility at Old Dominion University (ODU) under standard husbandry conditions. Mice of mixed genders and 8–10-weeks of age were used in this study. The Institutional Animal Care and Use Committee (IACUC) of ODU approved all the procedures. The animal protocol was approved under the ethics protocol number 24-009. All animals were housed in ventilated cages under controlled conditions, maintaining a standard temperature and humidity, and a 12-h light/dark cycle. Animals were provided with ad libitum access to standard food and water. To minimize animal suffering post-CEES exposure, all procedures were carried out strictly per e IACUC protocol 24-009. Animals were closely monitored for signs of distress, and humane endpoints were established, including weight loss exceeding 20%, abnormal behaviors, or inability to access food or water.

### 4.2. Animal Procedures

CEES-induced acute lung injury was modeled via the intratracheal (i.t.) injection of CEES (242640, Millipore Sigma, St. Louis, MO, USA) into mice at a dose of 6 mg/kg (50 μL) using a micro sprayer (Penn Century Inc. Wyndmoor, PA, USA) under anesthesia with ketamine and xylazine using well-established methods [28,44]. The control mice received 50 μL of PBS via i.t. injections. The CB2R agonist HU308 (3088, Tocris Biosciences, Minneapolis, MN, USA) was dissolved in a vehicle (1% DMSO + 1% Tween-80 in PBS). To activate CB2R, HU308 (1, 5, and 10 mg/kg, 100–200 µL) was administered to mice via the intraperitoneal (i.p.) route, 1 h before CEES administration and every 24 h after that. Age-matched wild-type (WT) mice of mixed genders, weighing 20–25 g, were assigned to the following groups: (1) vehicle + PBS, (2) HU308 + PBS, (3) vehicle + CEES (4) HU308 + CEES. Acute lung injury and inflammation were assessed at 24 h, 48 h, and 72 h after CEES exposure.

### 4.3. Measurement of Lung Mechanics

Lung mechanics were assessed using the FlexiVent system and FlexiWare software (SCIREQ, Montreal, Canada, version 8.0). Forty-eight hours after CEES exposure, mice were anesthetized with ketamine/xylazine and intubated intratracheally using a calibrated 18½ G catheter. The mice were ventilated at a low tidal volume (10 m L/kg) with a respiratory rate of 150 breaths per min for 10 min. Tissue dampening (G) and static lung compliance (Cst) were measured using FlexiWare software, following that previously described [45,46] and following the manufacturer’s guidelines. Briefly, the flexivent perturbation/ maneuver “quick prime” was used, which is a complex oscillation perturbation that provides an exhaustive assessment of the respiratory system at oscillatory frequencies well above and below the subject’s ventilation frequency. Tissue damping (G) reflects the amount of energy from the input test signal that is lost to heat within the tissues as a result of friction. It is closely related to tissue resistance and contains a contribution related to the resistance to airflow in the peripheral airways. Cst is an outcome extracted from the Salazar–Knowles equation and is calculated directly from the pressure–volume data of the PVs-P maneuver/perturbation in the FlexiWare software. Stepwise pressure–volume loops are typically used to assess static compliance.

### 4.4. Analysis of Bronchoalveolar Lavage Fluid (BALF)

Following the measurement of lung mechanics, the mouse lungs were lavaged with 3 mL of PBS (administered in three 1 mL aliquots). The total cell count in the BALF was determined using an automated cell counter (Countess II FL, ThermoFisher Scientific, Waltham, MA, USA). The total protein content in the BALF was quantified using the BCA Protein Assay Kit (Bio-Rad Laboratories, Hercules, CA, USA). Cytokine levels, specifically IL-6 and TNF-α, in the BALF were quantified via ELISA (R&D Systems, Minneapolis, MN, USA). Matrix metalloproteinase-9 (MMP-9) activity in the BALF was assessed through gelatin zymography as previously described [47] and following the manufacturer’s instructions. Equal volumes of BALF (20 μL) were loaded onto Novex™ 10% Zymogram Plus (Gelatin) Protein Gels, 1.0 mm (ZY00100BOX, ThermoFisher Scientific, Waltham, MA, USA), and electrophoresed for 90 min at 125 V. The gels were processed according to the manufacturer’s instructions: they were incubated in Zymogram renaturation buffer for 30 min with gentle agitation, followed by overnight incubation in Zymogram developing buffer at 37 °C. After incubation, the gels were stained with 0.25% Coomassie Brilliant Blue R-250 and subsequently de-stained. Clear bands were visualized and imaged using a gel documentation system (Bio-Rad Laboratories, Hercules, CA, USA).

### 4.5. Lung Tissue Processing

After collecting the BALF, the right mainstem bronchus was ligated with a 4-0 silk suture. The right lung was then excised, snap-frozen in liquid nitrogen, and stored at −80 °C for later analysis. The left lung was inflated with 4% paraformaldehyde (PFA) at 20 cm H2O pressure and fixed overnight at 4 °C. The left lobe was subsequently processed for hematoxylin and eosin (H&E) staining at the histology laboratory at EVMS to evaluate lung injury. The influx of immune cells was used to account for lung injury. Five images were taken from each section, and three to six mice per group were utilized in each experiment.

### 4.6. Isolation of Mouse Primary Alveolar Macrophages (AMs)

Mouse AMs were isolated as previously described [44,48]. After 48 h of exposure to CEES, the BALF was collected from the mice by lavaging the lungs with 3 mL of PBS, 1 mL at a time. The collected cell suspension was centrifuged at 1000 rpm for 10 min, at 4 °C, and the resulting pellet was resuspended in Dulbecco’s Modified Eagle’s Medium (DMEM) supplemented with 10% non-heat inactivated fetal bovine serum (FBS) (Mediatech, Inc., Manassas, VA, USA) and 1% penicillin–streptomycin (P/S) (Mediatech, Inc., Manassas, VA, USA).BALF cells were plated in a complete DMEM medium for 3 h, followed by extensive washing with PBS to remove unattached cells. The attached cells were identified as AMs. These cells were harvested by gentle scraping and subsequently used for the immunoblot analysis.

### 4.7. Immunoblot Analysis

Alveolar macrophages (AMs) or lung tissues were lysed using Pierce™ IP Lysis Buffer (ThermoFisher Scientific, Waltham, MA, USA). The lysates were centrifuged at 12,000 rpm for 15 min at 4 °C, and the supernatant was collected. The protein concentration in the supernatant was determined using the BCA Protein Assay Kit (Bio-Rad Laboratories, Hercules, CA, USA). For immunoblotting, 40 μg of total protein was resolved via SDS-PAGE and electroblotted onto a polyvinylidene fluoride (PVDF) membrane. The membrane was blocked with 5% non-fat milk in PBS containing 0.1% Tween-20 for 30 min at room temperature, followed by overnight incubation with primary antibodies at 4 °C. Afterward, the membrane was incubated with secondary antibodies and developed using ECL Western Blot Substrate (ThermoFisher Scientific, Waltham, MA, USA). The protein band intensity was quantified using ImageJ software, version 1.54 (NIH), with β-actin used as the housekeeping protein for normalization across all experiments. The primary antibodies used in this study were as follows: anti-iNOS (1:500, MAB9502, R & D Systems, Minneapolis, MN, USA), anti-Cox-2 (12282, 1:1000), anti-p65 (8242, 1:1000), anti-P-p65-Ser536 (3033, 1:500) from Cell Signaling Technology Inc., (Danvers, MA, USA) anti-arginase-1 (1:500, SC-271430, Santa Cruz Biotechnology, Dallas, Texas, USA), anti-4 Hydroxynonenal (4-HNE, 1:500, ab46545, Abcam, UK), and anti-β-actin (1:5000, A5441, Sigma-Aldrich, St. Louis, MO, USA).

### 4.8. Detection of Intracellular ROS

Cellular ROS production was measured using the DCFDA/H2DCFDA cellular ROS Detection Assay Kit (ab113851, Abcam, Cambridge, UK) as described previously [49] and following the manufacturer’s instructions. AMs were isolated from WT mice and cultured as previously described [44,48]. Fifty thousand cells were plated on a glass-bottom dish and incubated overnight in DMEM supplemented with 10% FBS and 1% penicillin–streptomycin (P/S). The cells were then pre-treated with vehicle or HU308 (5 μM) and exposed to CEES (50 μM) for 4 h in low-serum (0.5% FBS) DMEM media. After exposure, the cells were washed with wash buffer and incubated with a diluted DCFDA solution at 37 °C for 45 min. The cells were subsequently washed twice with wash buffer. ROS expression was observed and quantified using an Olympus IX73 fluorescent microscope, Tokyo, Japan and cellSense software, version 4.1.1.

### 4.9. Immunostaining

AMs were isolated from WT mice and cultured as previously described [44]. Fifty thousand cells were plated on a glass-bottom dish and incubated overnight in DMEM supplemented with 10% FBS and 1% penicillin–streptomycin (P/S). The cells were then pre-treated with vehicle or HU308 (5 μM) and exposed to CEES (50 μM) for 4 h in low-serum (0.5% FBS) DMEM media. After exposure, the cells were washed with PBS and fixed with 4% paraformaldehyde. Cells were stained for heme oxygenase-1 (HO-1), using an anti-HO-1 antibody (1:200, NBP1-31341: Novus Biologicals, Centennial, CO, USA), and for F4/80 (1:200, ab6640: Abcam, Cambridge, UK). The cells were subsequently incubated with species-specific secondary antibodies conjugated to fluorochromes, and the resulting fluorescence staining was captured using an Olympus IX73 fluorescence microscope.

### 4.10. Statistical Analysis

All results are presented as the mean ± standard error of the mean (SEM). Statistical comparisons were made using Prism 10.4.1 (GraphPad Inc., Boston, MA, USA). The group differences were analyzed using Student’s t-test or one-way ANOVA followed by Tukey’s post-hoc multiple comparison tests when appropriate. Differences were considered statistically significant at *p* < 0.05. All possible comparisons between study groups were systematically evaluated.

## 5. Conclusions

This study highlights the involvement of CB2R in regulating CEES-induced acute lung injury and inflammation in a mouse model. Our findings indicate that the pharmacological activation of CB2R significantly reduces CEES-induced pulmonary injury and inflammation. Specifically, CB2R activation by a selective synthetic agonist, HU308, suppresses proinflammatory macrophage responses and mitigates oxidative stress, thereby providing protection against CEES-induced lung damage. These results suggest that CB2R may offer a promising therapeutic target to counteract vesicant-induced pulmonary injury. One consideration in this study is the use of CEES, a structural analog of SM, to model SM-induced ALI instead of SM itself. Future studies using SM or advanced models could help validate and extend these findings.

## Data Availability

Data supporting the findings of this study are available from the corresponding author upon request.
